# Patient satisfaction and quality of vision after bilateral implantation of enhanced monofocal IOL and mini-monovision: a prospective study

**DOI:** 10.1186/s40662-025-00439-z

**Published:** 2025-06-16

**Authors:** Andrea Llovet-Rausell, Jorge Navalón-Tortosa, Vasyl Druchkiv, Javier Coloma-Bockos, Jaime Moya-Roca, Fernando Llovet-Osuna

**Affiliations:** 1 Clinica Baviera-AIER Eye Group, Valencia, Spain; 2 Chongqing Eye and Vision Care Hospital-AIER Eye Hospital Group, Valencia, Spain; 3 Department of Research and Development, Clinica Baviera-AIER Eye Hospital Group, Valencia, Spain; 4 Clinica Baviera-AIER Eye Hospital Group, A Coruña, Spain; 5 Clinica Baviera-AIER Eye Hospital Group, Madrid, Spain; 6 College of Medicine, Cardenal Herrera-CEU University, Valencia, Spain

**Keywords:** RayOne EMV IOL, Monovision, Enhanced monofocal intraocular lens, Patient satisfaction, Catquest-9SF, Patient-reported outcome measures, PROMs

## Abstract

**Background:**

Patient expectations for post-cataract surgery outcomes have risen. This study aims to evaluate patient satisfaction after bilateral implantation of enhanced monofocal IOL (RayOne EMV RAO200E) designed with positive spherical aberration, used for monovision with a 1.00 D offset.

**Methods:**

Prospective, non-comparative, interventional case series. Patients underwent bilateral cataract surgery and implantation of an enhanced monofocal IOL (RayOne EMV IOL RAO200E, Rayner, Worthing, UK) with target refraction of −1.00 D in the non-dominant eye and emmetropia in the dominant eye. Patient-reported outcome measures (PROMs) were assessed 3 months postoperatively using the Spanish version of the Catquest-9SF and a self-administered questionnaire. Other outcome measures included subjective refraction, visual acuity at various distances, and contrast sensitivity.

**Results:**

Both eyes of 51 patients were included (102 eyes). Three months postoperatively, all patients reported being satisfied or very satisfied with the overall surgical outcomes. The majority of patients reported that their vision during night driving was as good or better than before the surgery (95%); further, there was no difficulty in recognizing faces (93%), navigating uneven terrain (95%), and viewing prices while shopping (81%). The mean subjective spherical equivalent for dominant and non-dominant eyes were −0.24 ± 0.34 D and −0.86 ± 0.33 D, respectively. Binocular UDVA (4 m), UIVA (66 cm), and UNVA (40 cm) were 0.06 ± 0.09, 0.25 ± 0.12, and 0.30 ± 0.11 logMAR, respectively. Contrast sensitivity was within the population norms (CSV-1000).

**Conclusion:**

Monovision with the RayOne EMV IOL provided high patient satisfaction, with preserved contrast sensitivity, good distance vision, and functional intermediate and near vision.

*Trial registration*: Clinicaltrials.gov, NCT06528678. Registered 22 July 2024—Retrospectively registered, https://clinicaltrials.gov/study/NCT06528678.

## Background

With the rapid advancement of intraocular lens (IOL) technologies, patient expectations have also increased in terms of their desired outcomes after cataract surgery [1]. A well-known fact is that patients value spectacle independence [2], and there is a growing recognition of the importance of functional vision [3] which refers to good visual acuity across a range of distances necessary for daily activities. This has led to the development of presbyopia-correcting optics such as extended depth-of-focus (EDOF) and trifocal IOLs, providing good distance vision with improved near and intermediate vision [4]. However, dissatisfaction is still observed with these IOLs, mainly relating to lowered visual quality due to the presence of photic phenomena and loss of contrast sensitivity, even necessitating IOL exchanges in the most severe of cases [5–7].

Therefore, alternative presbyopia-correcting strategies are needed to provide maximal patient satisfaction by combining an extended range of vision with good visual quality. One such method is the monovision strategy, which optimizes one eye for distance vision and the other eye for near. Conventional monovision with monofocal IOLs has been found to achieve a range of vision comparable to that of multifocal IOLs while preserving the quality of vision [8]. However, the large anisometropia of conventional monovision can reduce stereopsis and cause asthenopia [9–11], which can lead to patient dissatisfaction [12]. Additionally, monovision with monofocal IOLs creates a blur zone in the intermediate distance range, compromising intermediate vision [13]. Monovision with multifocal and EDOF IOLs has been attempted to further improve intermediate and near vision results [14, 15]; however, the diffractive optics adopted by some of these lenses still produced unwanted photic phenomena [14, 15], known to affect activities such as nighttime driving [16]. This can decrease satisfaction with visual outcomes as several studies have shown dysphotopsia to be a significant cause of patient dissatisfaction after cataract surgery [17–19]. Although, refractive IOLs, unlike diffractive designs, cause minimal dysphotopsia, they are associated with a lower depth of focus that may also contribute to decreased patient satisfaction [20].

A potential solution to the above limitations is the use of monovision with enhanced monofocal IOLs, which is a new generation of IOLs that achieve better depth of focus compared to standard monofocal lenses [21]. The RayOne EMV RAO200E (Rayner, Worthing, UK) is a non-diffractive enhanced monofocal IOL that utilizes positive spherical aberrations to increase the range of vision without inducing the photic phenomena seen with diffractive multifocal and EDOF IOLs [22, 23]. It was hypothesized that the increased range of vision could enhance interocular fusion in monovision set-up by creating a broader blended zone between both eyes, improving intermediate vision without compromising stereoacuity [13]. This avoids the side effects of anisometropia, such as asthenopia, while providing spectacle independence and a high contrast sensitivity, potentially leading to higher patient satisfaction.

According to the manufacturer, the RayOne EMV RAO200E IOL is specially optimized for use in a monovision configuration, where one eye is targeted for distance vision with a range from −0.25 to 0.25 D and the other eye is targeted in a range from approximately −0.75 to −1.25 D. While previous peer-reviewed clinical studies using bilateral emmetropic targets with the RayOne EMV obtained good contrast sensitivity [22] and good visual acuity at distance [22, 23] intermediate [23] and near [22] the performance of this IOL in a monovision configuration has not yet been studied clinically.

The concept of modified monovision or mini-monovision was introduced to overcome the side effects of monovision, mentioned above. Unlike conventional monovision with an offset of approximately 2.50 D, mini-monovision entails an offset of 0.75–1.25 D. This ensures a larger area of interaction between the range of vision of both eyes causing fewer side effects, excellent distance vision, and functional intermediate vision [24, 25].

In this study, we investigated patient satisfaction after bilateral implantation of the RayOne EMV RAO200E enhanced monofocal IOL for mini-monovision with an offset of 1.00 D.

## Methods

### Ethics and study cohort

This prospective, interventional, non-comparative study was performed at the Clinica Baviera-AIER Eye Hospital Group, Spain. The study was reviewed and approved by the Ethics Committee of the Hospital Clinico San Carlos (Reference: 21/301-O_P) and adhered to the tenets of the Declaration of Helsinki. Written informed consent was obtained from all the patients preoperatively.

We included patients aged ≥21 years who were diagnosed with bilateral cataracts. Other inclusion criteria were preoperative corneal astigmatism <1.00 D, potential for corrected distance visual acuity (CDVA) of 0.18 logMAR or better postoperatively and calculated IOL power in the range of 10.00–30.00 D. Subjects were excluded if they had ocular comorbidities or conditions that could affect the outcome. Eyes with previous corneal refractive surgery were excluded.

Sensorial dominance was tested using both the hole-in-card and 1.00 D plus tests.

### Visits

The patients attended a preoperative visit, a surgical visit for each eye, and a postoperative visit three months after surgery.

### Intraocular lens

The RayOne EMV RAO200E lens (Rayner, Worthing, UK) is a single-piece, non-diffractive, aspheric, enhanced monofocal IOL made of Rayacryl hydrophilic acrylic, with a refractive index of 1.46 and an Abbe number of 56 [26]. The optical design induces controlled positive spherical aberration in the IOL (maximum 0.15 µm across the 6-mm optic) to elongate the focal range from far into intermediate [23]. Owing to its optic design, the IOL appears identical to a standard monofocal IOL, without zones or rings, thereby potentially avoiding contrast loss [22] or photic phenomena [23].

The RayOne EMV IOL has a total length of 12.5 mm and an optical diameter of 6.0 mm. Other key features include the Amon-Apple 360° enhanced square edge to prevent lens epithelial cell migration and posterior capsule opacification (PCO), anti-vaulting haptic (AVH) technology for lens stability [27], and delivery via a fully preloaded RayOne injector with a syringe-shaped design, which allows one-handed IOL implantation through a 2.2-mm corneal incision.

The power range of the IOL extends from 10.00 to 30.00 D in 0.50 D increments.

### Surgical procedure

The surgeon’s preferred micro-incision technique was used to perform standard phacoemulsification through a 2.2-mm corneal incision under topical anesthesia.

Biometry was performed using an IOLMaster500 (Carl Zeiss Meditec AG, Jena, Germany). The Barrett Universal II [28], (Lens Factor 1.67; Design Factor 3.5), and the Kane Formula [29], were used to calculate the IOL power. The target refraction was emmetropia in the dominant eye and −1.00 D in the non-dominant eye for a mini-monovision setup.

### Postoperative assessments

Patient-reported outcome measures (PROMs), including patient satisfaction, visual difficulties, and spectacle independence, were assessed at 3 months post-surgery using the Spanish version of the Catquest-9SF [30]. It is a clinically validated questionnaire that focuses on patient-reported outcomes regarding visual function and activity limitation in daily life. The response “cannot decide” was treated as missing data. Each patient’s score was transformed into a Rasch-calibrated score. Additionally, a self-administered questionnaire was used to assess night vision, night driving, satisfaction with the overall surgical outcome, and whether the patient would choose the same surgery again.

Subjective refraction was also assessed. Visual acuity was measured under photopic conditions (85 cd/m^2^) with 100% contrast using ETDRS charts and was reported in logMAR. Monocular and binocular uncorrected and corrected distance visual acuity (UDVA and CDVA, respectively) were measured at 4 m. Uncorrected intermediate visual acuity (UIVA) was measured monocularly and binocularly at 66 cm. Uncorrected near visual acuity (UNVA) was measured monocularly and binocularly at 40 cm.

Contrast sensitivity was measured in log units using the CSV-1000 chart (Vector Vision, Greenville, OH, USA) at spatial frequencies of 3, 6, 12, and 18 cycles per degree (CPD).

Adverse events were recorded during all visits.

### Statistical analysis

Data analysis was performed using Excel software for Windows (Microsoft, Redmond, WA, USA). The study data were analyzed using descriptive statistics, including the mean and standard deviation (SD) for each parameter.

## Results

A total of 51 patients (27 females and 24 males), mean age of 72.67 ± 7.41 years, were bilaterally implanted (102 eyes) with the RayOne EMV IOL. All patients reached the 3-month follow-up. Table 1 shows the mean preoperative demographic data.

**Table 1:** Patient demographics

Population	*n* (%)
Sex	
Female	27 (53%)
Male	24 (47%)
Dominant eye	
Right eye	30 (59%)
Left eye	21 (41%)

Item	Mean ± SD	Range
Age (years)	72.7 ± 7.4	47–88
SEQ (D)	0.91 ± 3.05	−11.25–5.00
Binocular CDVA (logMAR)	0.25 ± 0.15	0.60–0.00
IOL power (D)	22.05 ± 3.38	10.00–28.00
Mean keratometry (D)	43.92 ± 1.45	41.00–47.25
Axial length (mm)	23.27 ± 1.21	21.18–28.08
Anterior chamber depth (mm)	3.02 ± 0.40	1.97–4.06
IOP (mmHg)	17.1 ± 3.1	11.0–24.0

*SD* = standard deviation; *SEQ* = spherical equivalent; *CDVA* = corrected distance visual acuity; *IOL* = intraocular lens; *IOP* = intraocular pressure

### Questionnaire outcomes

All patients reported being satisfied or very satisfied with the overall outcome of the surgery, and 100% of the patients reported that they would choose the same surgery again (Table 2). In terms of quality of vision, 95% of the patients reported driving as well or better than before the surgery, and 95.8% reported that their night vision was the same or better than before the surgery.

**Table 2:** A self-administered questionnaire to assess patient satisfaction regarding night vision

Evaluate your night driving after the procedure	
At night I drive as well or better than before the operation	95%
At night I drive a little worse than before the operation, but it is not a problem	5%
At night I drive much worse than before the operation and I feel insecure	0%
I have stopped driving because I feel very insecure	0%

Evaluate your night vision after the procedure	
At night my vision is the same or better than before	95.8%
At night my vision is somewhat worse than before	2.1%
At night my vision is much worse than before	2.1%

Considering all aspects of the overall outcome of the operation, I am	
Very satisfied with the result	85.4%
Fairly satisfied with the result	14.6%
Fairly dissatisfied with the result	0%
Very dissatisfied with the result	0%

I would operate again with the same technique	
Yes	100%
No	0%

**Fig. 1:**
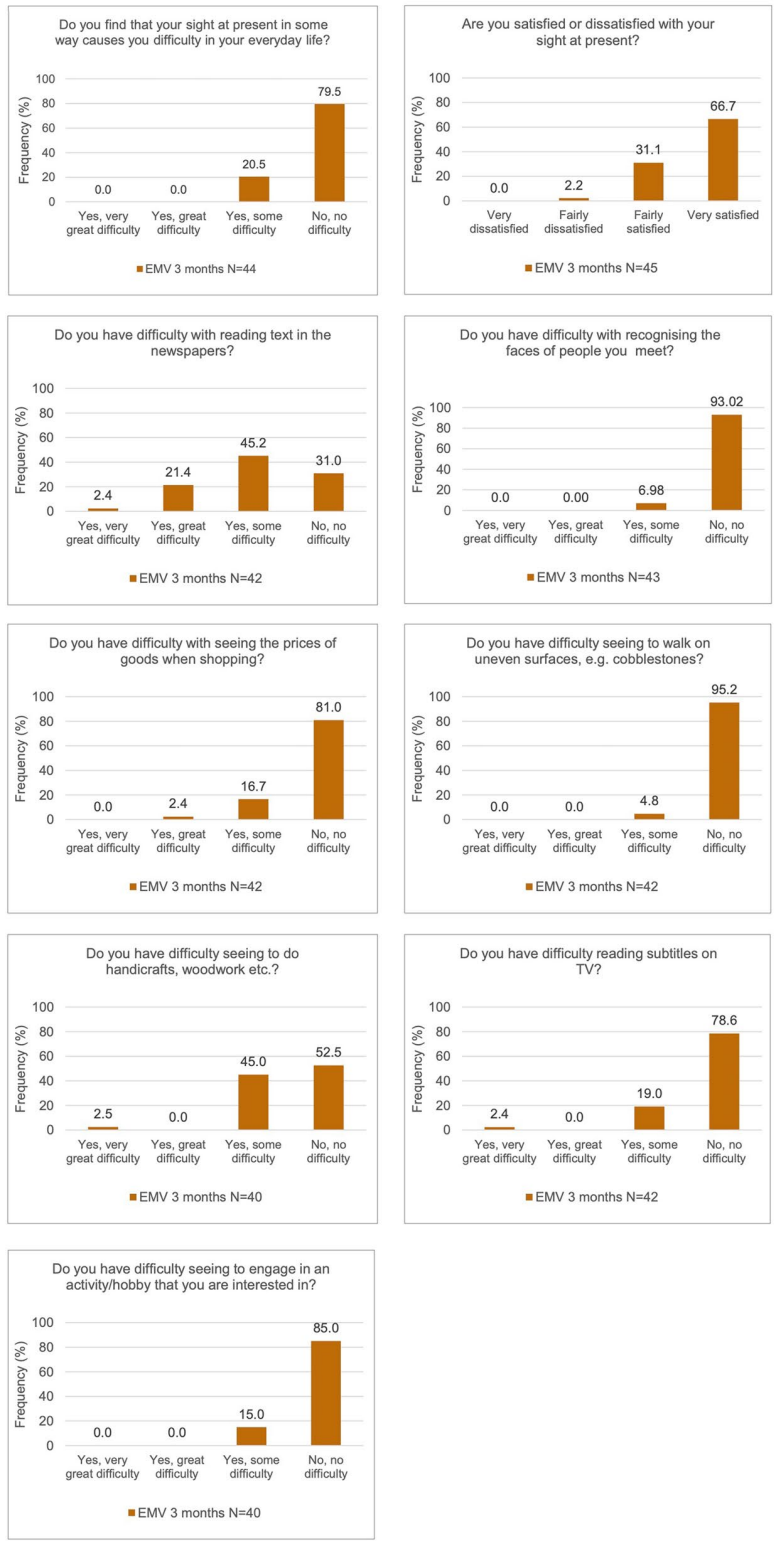
Results of the Catquest-9SF questionnaire

Figure 1 illustrates the findings of the Catquest-9SF questionnaire. A significant percentage of patients (97.8%) expressed themselves being either very satisfied or fairly satisfied with their current vision. The vast majority of patients also expressed themselves having no difficulty in their everyday life because of their sight (79.5%), navigating uneven terrain (95.2%), recognizing faces (93%), engaging in a hobby (85%), reading text on television (78.6%), and viewing prices while shopping (81%). One-third of the patients reported no difficulties in reading newspapers. Half of the patients reported no difficulty in seeing the handicrafts.

The overall score for Rasch analysis was −3.09 ± 0.68. When analyzing each question, we found that the mean value for difficulties in their life was −3.42 ± 1.11 and −1.57 ± 1.43 for the satisfaction with their actual vision. For tasks involving distance vision such as recognizing faces or watching TV subtitles, the average score was −3.44 ± 0.70 and −3.75 ± 1.81, respectively. For near-vision tasks, such as viewing prices or doing handicrafts, the mean values were −3.86 ± 1.26 and −1.97 ± 1.65. “Practicing a hobby” obtained a mean Rasch score of −4.58 ± 0.88.

### Refractive results

The mean absolute difference between dominant and non-dominant eyes per patient at 3 months was 0.79 ± 0.25 D (0.25–1.50 D). In the non-dominant eyes, postoperative mean spherical equivalent (SEQ) was −0.86 ± 0.33 D (−1.63 to 0.13 D), while in the dominant eyes, SEQ was −0.24 ± 0.34 D (−1.13 to 0.50 D). In the dominant eyes, 86.3% of eyes were within ±0.50 D of target refraction, and 96.1% of eyes were within ±1.00 D of target refraction, while in the non-dominant eyes, 86.3% of eyes were within ±0.50 D of target refraction, and 98.0% of eyes were within ±1.00 D of target refraction.

### Visual acuity results

**Fig. 2:**
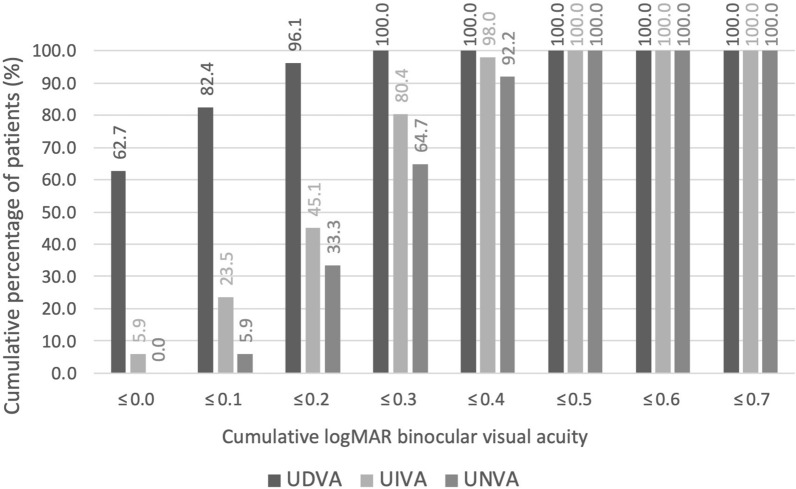
Distribution of binocular UDVA, UIVA, and UNVA at the 3-month postoperative visit. UDVA, uncorrected distance visual acuity; UIVA, uncorrected intermediate visual acuity; UNVA, uncorrected near visual acuity

At 3 months postoperatively, the mean monocular UDVA was 0.10 ± 0.13 logMAR in the dominant eyes targeted for emmetropia, while the mean binocular UDVA was 0.06 ± 0.09 logMAR (Table 3). The mean binocular UIVA was 0.25 ± 0.12 logMAR and mean binocular UNVA was 0.30 ± 0.11 logMAR. All the eyes (100%) had a monocular CDVA of 0.2 logMAR or better. Binocular UDVA was 0.1 logMAR or better in 82.4% of patients, binocular UIVA was 0.3 logMAR or better in 80.4% of eyes, and binocular UNVA was 0.3 logMAR or better in 64.7% of eyes (Fig. 2).

**Table 3:** Binocular and monocular (dominant eye) uncorrected distance visual acuity (UDVA) and corrected distance visual acuity (CDVA), uncorrected intermediate visual acuity (UIVA), uncorrected near visual acuity (UNVA) at 3 months postoperatively

Visual acuity (logMAR)			Preoperatively	3 months postoperatively
*Patients (n* = *51)*				
CDVA	Binocular	Mean ± SD	0.25 ± 0.15	0.03 ± 0.05
		Range	0.00–0.60	0.00–0.20
UDVA	Binocular	Mean ± SD	0.73 ± 0.37	0.06 ± 0.09
		Range	0.10–1.40	0.00–0.30
UIVA (66 cm)	Binocular	Mean ± SD	0.75 ± 0.18	0.25 ± 0.12
		Range	0.20–1.10	0.00–0.50
UNVA (40 cm)	Binocular	Mean ± SD	0.78 ± 0.25	0.30 ± 0.11
		Range	0.20–1.40	0.10–0.50
*Dominant eye (n* = *51)*				
CDVA	Monocular	Mean ± SD	0.30 ± 0.21	0.03 ± 0.06
		Range	0.00–1.00	0.00–0.20
UDVA	Monocular	Mean ± SD	0.80 ± 0.38	0.10 ± 0.13
		Range	0.05–1.40	0.00–0.50

### Contrast sensitivity

**Fig. 3:**
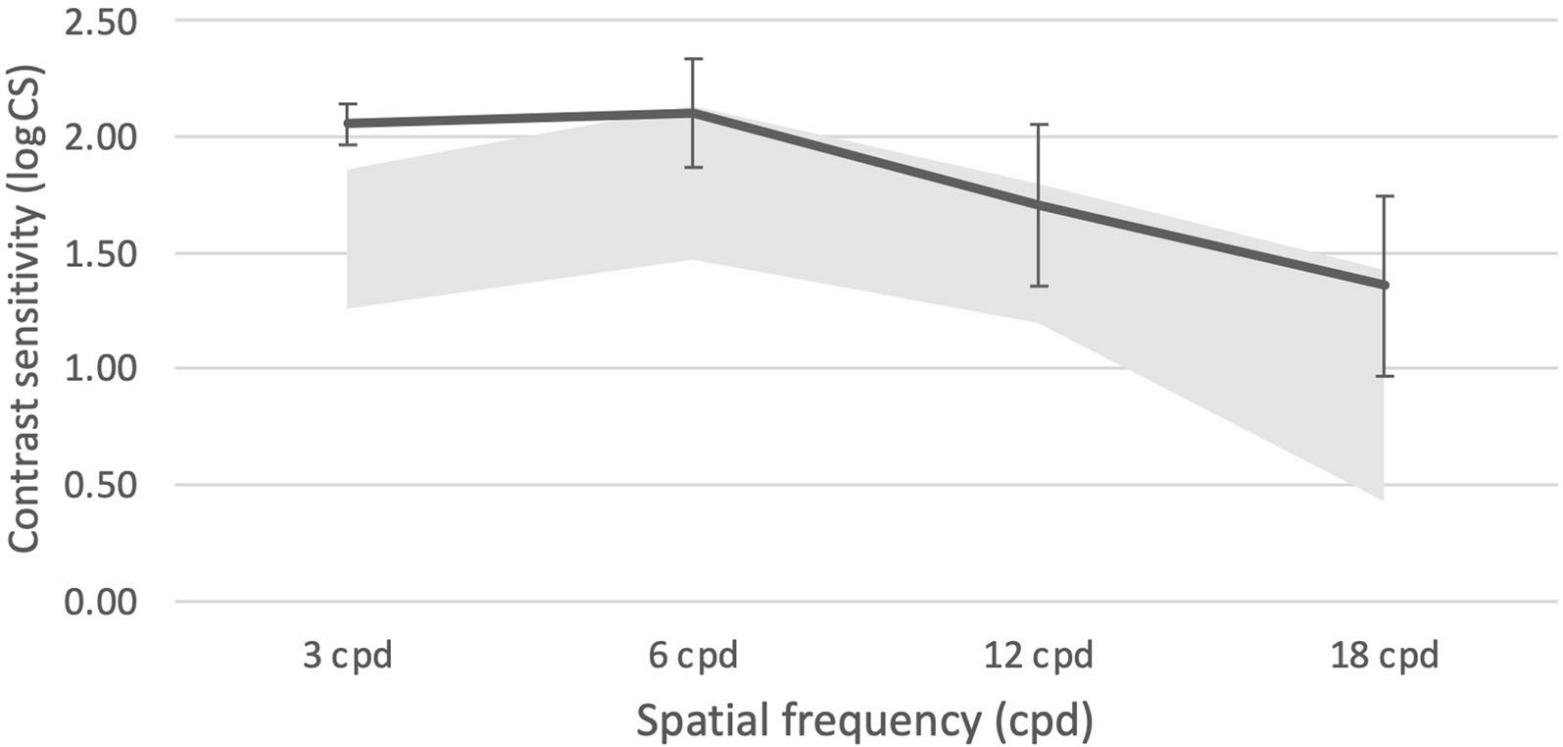
Binocular contrast sensitivity at photopic conditions. The bold line represents the mean value for the study while the grey area represents normal limits

Contrast sensitivity at 3, 6, 12, and 18 CPD were 2.05 ± 0.09, 2.10 ± 0.23, 1.70 ± 0.35, and 1.36 ± 0.39, respectively. Contrast sensitivity values approached the upper level of the population norms for the CSV-1000 (Fig. 3).

### Adverse events

No intra- or postoperative adverse events or asthenopia symptoms were reported.

## Discussion

To our knowledge, this is the first study assessing patient satisfaction and quality of life with the RayOne EMV IOL in a monovision setup. Here, the RayOne EMV IOL achieved good results in terms of patient satisfaction in a private clinic setting: 97.8% of patients were either very satisfied or fairly satisfied with their vision, and 100% would choose to undergo the procedure again with the same technique. Additionally, more than two-thirds of patients reported no difficulties due to their eyesight in everyday life and one-third did not have difficulties with near-vision tasks like reading newspapers and doing handicrafts. High patient satisfaction was seen for daily tasks requiring intermediate vision, such as recognizing faces, reading price tags while shopping, or reading subtitles on television. These could have contributed to the high overall patient satisfaction observed in our study.

Another explanation for the high satisfaction seen in our study might be accredited to the good visual quality achieved by the RayOne EMV IOL. Difficulty in night driving has been shown to be associated with reduced contrast sensitivity and the presence of dysphotopsia [31, 32]. Thus, the high patient satisfaction with night vision (95.8%) and night time driving (95%) observed can likely be attributed to the preserved contrast sensitivity, which was within age norms [33], and the lack of photic phenomena with the RayOne EMV IOL [28]. In a study by Stock et al. comparing bilateral multifocal IOL implantation and monovision with standard monofocal IOL [34], the proportion of patients reporting no difficulty with night vision (75% and 96.5%, respectively) and night driving (37.5% and 86.2%, respectively) were lower than that observed in our study.

Overall patient satisfaction with vision (97.8%) and surgery (100%) observed in our study were comparable to or better than those observed for monovision with other bifocal or EDOF IOLs [14, 15, 35]. Furthermore, these IOLs were associated with visual symptoms due to photic phenomena as a result of their diffractive or wavefront-shaping optics.

In a study on a wavefront-shaping EDOF IOL (AcrySof IQ Vivity, Alcon Inc.), monovision with −0.75 D offset resulted in 84% of patients being completely or mostly satisfied with their vision without glasses. Yet, dysphotopsia was reported, with 24% of patients experiencing moderate or severe glare, and 33% experiencing moderate or severe halos [35]. Similarly, Kohnen et al., reported that 25% of patients had halos and glare after implanting the same IOL with a target of emmetropia in both eyes [36]. In the CONCERTO study comparing a diffractive extended range of vision IOL (Tecnis Symfony, Johnson & Johnson Vision Inc.) in monovision and non-monovision settings [15], only 92% of patients in the monovision group reported that they would choose the same IOL again compared to the 100% in our study. However, in the CONCERTO study, dysphotopsia was reported by 13% of patients in the monovision group being moderate to severe halos and 4% experienced moderate to severe glare. Another study on monovision with the same IOL found 32% of patients to have moderate to severe dysphotopsia [37]. In a study by Chang et al. on monovision with a diffractive multifocal IOL (Tecnis ZMB00 IOL, Abbott Medical Optics, Inc.) [14], 52% of patients reported halo while 29% patients reported glare in their study.

It has been established that monovision itself can cause night driving difficulties due to reduced interocular blur suppression in scotopic conditions [10], and this can be compounded by the dysphotopsia associated with multifocal IOLs that are also known to independently interfere with night time driving [34]. The high satisfaction rates for night vision and night driving observed in our study suggest that monovision with the RayOne EMV IOL provides good-quality vision.

The large anisometropia of conventional monovision can cause asthenopia, reduced contrast sensitivity, and loss of stereopsis [9–11]. In our study, no asthenopia symptoms were reported by patients and contrast sensitivity was maintained within normal limits. The majority of patients (95.2%) had no difficulty walking on uneven surfaces, which requires good binocular vision [38], suggesting that binocular stereoacuity was maintained. Hence, monovision with RayOne EMV and an offset of 1.00 D produced no side effects that are seen with monovision with standard monofocal and diffractive lenses.

Mini-monovision aims to provide an adequate range of vision [39]. Patient expectations for cataract surgery outcomes include good intermediate vision because of increased usage of digital devices [22, 40]. The high patient satisfaction observed for intermediate distance range activities in our study suggests that satisfactory functional vision was achieved by mini-monovision with the RayOne EMV IOL.

RayOne EMV IOL was designed to induce controlled positive aberration of up to 0.15 μm across its 6 mm optic, complementing the naturally occurring corneal spherical aberration [23, 41]. Positive spherical aberrations have been shown to increase the depth of focus by up to 2.00 D [42], creating a single elongated focus that provides a continuous range of vision rather than the multiple discrete foci seen in multifocal IOLs. However, the effect of spherical aberration is highly pupil-dependent [43], as the pupil controls the portion of peripheral rays entering the eye, subjected to the effect of spherical aberration.

Optical bench studies have demonstrated that the modulation transfer function (MTF) value for the RayOne EMV IOL was superior when used on smaller pupils but declined with wider pupils. This suggested that far vision quality may depend on pupil size while closer distances benefit from reflex pupil constriction [44]. Despite this, patient reported outcomes in the current study indicated high satisfaction with tasks requiring distant vision and nighttime driving. This is likely due to the benefits of binocular vision and the mini-monovision setting.

According to the manufacturer, the expected range of vision for the RayOne EMV IOL is 1.25 D [44]. With binocular vision with a 1.00 D offset, it is expected to increase to 2.25 D [45]. In a study evaluating the RayOne EMV IOL, the defocus curve demonstrated a distance-corrected monocular visual acuity of 0.2 logMAR or better between 0.50 and −0.75 D when emmetropia was targeted in both eyes. This range improved with distance-corrected binocular vision extending to 1.00 and −1.25 D [23].

The limitations of this study include a small sample size, a relatively short follow-up period, and a lack of a comparison group consisting of other IOL optic designs, such as EDOF or multifocal IOLs. Preoperative corneal aberrations should have been measured to mitigate the potential negative impact on visual outcomes, particularly if postoperative spherical aberrations exceeded 6 μm [42]. Evaluating the defocus curve could have provided valuable insights into the range of vision offered by the IOL in a mini-monovision setting. Assessing patient-reported photic phenomena and measuring stereoacuity could also have aided in direct comparisons of outcomes with other studies. Future comparative studies and randomized trials can provide further evidence on the efficacy of monovision with the RayOne EMV IOL in comparison to other presbyopia-correcting strategies. Finally, this study acknowledges the limitation of conducting contrast sensitivity measurements using the CSV-1000 with a single repetition. While this approach reflects real-world clinical conditions, it may reduce the statistical reliability of the results.

## Conclusion

In summary, 1.00 D monovision with the RayOne EMV IOL led to a very high level of overall satisfaction, with high satisfaction for daily tasks requiring intermediate or near vision due to functional visual acuity across all distances. Additionally, it maintained a normal range of contrast sensitivity.

## Data Availability

The datasets used and/or analyzed during the current study are available from the corresponding author on reasonable request.

## Abbreviations

AVH: Anti-vaulting haptic
CDVA: Corrected distance visual acuity
CPD: Cycles per degree
EDOF: Extended depth-of-focus IOL
IOL: Intraocular lens
PCO: Posterior capsule opacification
PROMs: Patient-reported outcome measures
SD: Standard deviation
UDVA: Uncorrected distance visual acuity
UIVA: Uncorrected intermediate visual acuity
UNVA: Uncorrected near visual acuity
